# Intimate geographies of virginal blood

**DOI:** 10.1177/14744740221110586

**Published:** 2022-07-08

**Authors:** Elisabeth Militz

**Affiliations:** University of Guelph, Canada

**Keywords:** geographies of the body, heterosexuality, Instagram, intimate justice, Kyrgyzstan, marriage, resistance, virginity

## Abstract

Feminist scholars, activists, and artists have long addressed the topic of virginity and have dismantled it as a powerful, globally circulating, and gendered myth. It affects how many woman-identifying people experience how their bodies become (a)sexual. Centrally, the myth of virginity has been shown to be mobilized in support of colonial, ethnonationalist identity projects. In Kyrgyzstan, disciplining women through policing their sexual behavior co-constituted nation-building projects after the dissolution of the Soviet Union. Drawing on data collected since 2017 (qualitative interviews in Bishkek and Osh and qualitative research on Instagram), I examine intimate geographies of the virginity myth in Kyrgyzstan. Building on geographic scholarship on intimacy and body parts, I discuss the ways in which virginal blood works both to submit to and to reclaim one’s intimate body spaces and sexual practices. I argue that people affected by the virginity myth create the foundations for intimate justice in Kyrgyzstan. On the one hand, people are reclaiming authority over their intimate bodies through subverting sexist systems of sexual control and through expanding discursive horizons of virginity performances. On the other hand, activists on Instagram are supporting digital public spaces that allow to author virginal blood narratives and provide resources on intimate body knowledge. Analyzing the scalar doings of virginal blood, the paper contributes a case study on intimate justice and the geographies of the body in Kyrgyzstan. My analysis encourages further examination of the capacities of the body to better understand how certain body parts turn into key sites of (geo)political struggles and can transform them.

## Virginity power

*If you want to be happy for the rest of your life with your husband, you should bleed [on the wedding night]. It doesn’t matter how, even if you had [sexual] experience before marriage.* ^1^

I meet 26-year-old Bermet^2^ in Osh, Kyrgyzstan, in her office at a local youth organization in August 2017. She talks about the social expectation for women to prove virginity in the heterosexual wedding night through producing drops of blood on a white sheet – a common expectation there and in other places across Eurasia.^3^ Bleeding on the wedding night demonstrates the ability to perform dominant ideals of femininity and masculinity^4^ and thus promises future happiness, as Bermet points out. Her words hint at both the stability and subversion of virginity performances that are ‘practices glorifying young women’s premarital virginity’.^5^ While social norms for female premarital virginity persist in various communities worldwide,^6^ women^7^ in Kyrgyzstan also follow different strategies to produce the blood expected on the wedding night^8^ – after all, ‘it doesn’t matter how’ a person performs this symbol of virginity, since it is the drops of blood that confirm a bride’s virginal status.

Starting my inquiry from the key role virginal blood plays in virginity performances, the aim of this paper is to examine the intimate geographies of virginal blood that reveal the different scales on which virginal blood operates including the digital spaces virginal blood enables. Building on intimate geographies^9^ and geographic scholarship on blood,^10^ my analysis reconstructs the appropriation and the scalar doings of virginal blood. Acknowledging the virginity myth’s inherent intimate injustice, my analysis seeks to understand what geographers and cultural studies scholars can learn about the building of intimate body justice from focusing on virginal blood.

Political theorist Shatema Threadcraft provides a comprehensive account of intimate (body) in/justice.^11^ Drawing on the experience and knowledge of Black women in the United States, her work on ‘intimate justice traces the long and still incomplete struggle for [. . .] the free and equal use of the powers and capacities of the black female body’.^12^ In this paper, I follow Threadcraft’s invitation as ‘any system that allowed the longstanding exclusion of a specific group of women from state protection for women’s bodily integrity and bodily health, for example, would merit intimate justice’,^13^ and examine the resistance to the control of the female body through the myth of virginity in Kyrgyzstan.

After all, through the expectation to perform virginity, women in Kyrgyzstan experience intimate injustice when they are denied sexual privacy and intimate self-determination. As a social norm, the virginity myth is mobilized to regulate and constrain certain bodies’ sexual practices and experiences. State institutions fail to provide both protection from virginity-myth-induced violence and fail to provide knowledge about sexuality for women. Building on Threadcraft’s groundbreaking project toward intimate justice and her focus on women’s resistance to intimate injustice, I argue that the foundations for intimate justice in the context of the virginity myth in Kyrgyzstan are built through different ways of appropriating virginal blood that lead to reclaiming the wedding night’s intimate spaces, the sexual body, and digital community building around intimate body knowledge.

Feminist scholars and activists across different parts of the world have long dismantled virginity as a gendered myth that affects how women experience how their bodies become (a)sexual.^14^ Virginity performances in the heterosexual wedding night and the taboo of sexual experiences for unmarried women signify the virginity myth as a patriarchal, sexual norm that defines and demarcates an ‘existence in the world as embodied, sexual subjects’^15^ for many women. It is crucial, though, to acknowledge the different valuations of virginity performances. French philosopher Luce Irigary, for example, considers virginity to have spiritual potential beyond its misogynist connotations as ‘a feminine in-dwelling’.^16^ And while many Western-educated feminists consider virginity a ‘malaise which stems from sexual inequality’,^17^ some African feminists, such as Sinenhlanhla Chisale, emphasize the empowering function of the virginity myth. Chisale understands virginity as ‘a hidden patriarchal tool of power but also as a hidden feminine tool used by women to claim authority to their sexual bodies’.^18^

The heterosexualization of the virginity myth also plays out in its racialized dimension. Anne McClintock demonstrates ways in which the myth of virginity comes to justify and legitimate colonial entitlements to territory and people in the context of European imperialism in parts of Africa. Here, the myth of virginity functions to eliminate both land and humans:

*Within colonial narratives, the eroticizing of “virgin” space also effects a territorial appropriation, for if the land is virgin, colonized peoples cannot claim aboriginal territorial rights, and white male patrimony is violently assured as the sexual and military insemination of an interior void.*^19^

In the colonial present, and as a form of resisting the racialization of the virginity myth, some racialized women also appropriate the myth of virginity. For example, women of Maghrebine origin in France challenge everyday racialization regarding their sexual practices by the dominant French population through ‘adher[ing] to the norm of virginity on their own initiative’.^20^ This shows how virginity myths root in ‘social discourse and imagination [and not] in any biological reality’.^21^ After all, the correlation between drops of blood and first vaginal penetration is scientifically false.

Geographers have so far not engaged with virginity myths or virginal blood although the spatial and scalar implications of the social institutionalization of virginity myths^22^ holds great potential. Anthropologist Ayse Parla, for example, has shown the virginity myth’s complicity in nation-building projects. For the case of Turkey, she reconstructs how virginity tests are ‘emblematic of the incorporation of the preoccupation with women’s modesty [. . .] into the mechanisms of surveillance deployed by the modern [nation] state’.^23^ Similarly, historical work has illustrated how evangelicals in the United States, for example, propagate ‘a rhetoric of sexual purity, or more precisely a rhetoric of sexual fear’^24^ that links premarital virginity to national prosperity and global security. Sociologist Elena Kim’s work in the context of Kyrgyzstan demonstrates the significance of virginal blood’s symbolic and material qualities for these spatial identity projects. She has shown that virginity performances involving ‘enough blood’^25^ legitimize gendered ethno-nationalist ideas. Performing virginity during the wedding night is crucial for the ‘new Kyrgyz woman[’s] [. . .] re-establishment of womanly features seen as erased during the long Soviet era and under the continuous new threat of globalization’.^26^ My analysis of virginal blood builds on this entanglement between ethno-nationalisms and the institutionalization of the virginity myth.

In what follows, I revisit the intimate geographies of blood to lay the conceptual foundations for my analysis. I then provide more detail on the empirical research informing the analysis before contextualizing the intimate injustices legitimized through virginity myths in Kyrgyzstan. I uncover the scalar doings of virginal blood in two steps. First, I analyze different ways in which virginal blood is appropriated to transform public intimate spaces of the body and the family. Second, I turn to digital spaces of sexuality knowledge on Instagram to show ‘intimate-sphere resistance efforts’^27^ in the digital public. I conclude with a discussion on ways toward intimate justice in Kyrgyzstan.

## Understanding intimate geographies of virginal blood

Geographers have long identified the scale of the intimate as crucial site for (geo)political and cultural analyses to draw attention to unjust power and social relations.^28^ Global intimate entanglements of (geo)political projects^29^ illustrate how households, family relations, or bodies become central sites of negotiation for border demarcations,^30^ nationalisms,^31^ and migration policies.^32^ In today’s digital era, the everyday production, transformation, and appropriation of digital spaces illustrates how intimacy in the sense of sharing knowledge, loving, and caring for one another stretches ‘beyond the boundaries of the domestic’.^33^ Intimate geographies then ‘illuminate the connectedness and blending of spaces and scales within people’s lived, embodied and emotional experiences’.^34^

Bodies and body parts remain key sites for intimate geographical analyses. The attention to bodies helps to understand ‘how power acts spatially in the world to control, regulate, confine, produce, construct, delimit, gender, racialize, and sex the body’.^35^ The body and its materials such as hair, fat, or the placenta become the intimate scale where postcolonial, racist, gendered, and nationalist politics unfold and are contested.^36^

Blood provides a particularly interesting example for analyzing the scalar doings of bodily materials. Blood is an essential bodily fluid which transgresses and challenges bodily boundaries.^37^ At the same time, ‘blood has socio-cultural, scientific and commercial value’.^38^ For example, blood is believed central for questions of kinship forming the foundations for racialized nation-building projects.^39^ Scientific interests in blood range from technological developments in biomedicine to legal examinations of liability in cases of blood contamination.^40^ Indeed, blood has long turned into a central commodity in the global ‘bio-resource economy’.^41^ Yet not all blood is the same. Powerful hierarchies between differently gendered, sexualized, racialized and commodified types of blood regulate access to and comfort across public and intimate spaces.^42^

Current geographical analyses of blood, however, remain incomplete as they do not address virginal blood. Yet, virginal blood is remarkable as it operates across different scales and functions through various mechanisms of power at the same time. Virginal blood is gendered. Only women are held responsible for its absence/presence. After all, the absence of blood during virginity performances calls her virginity into question, not his.^43^

Virginal blood is thus also sexualized. Through its connection to heterosexual marriage practices, virginal blood naturalizes and reinforces heterosexuality as social norm. The ‘“myth of blood” that connects disparate members of the family both nuclear and national’^44^ validates the heterosexual connection between bodies as the only ‘natural foundation of the social’.^45^

The sexualization of virginal blood indicates its racialization. While virginity myths persist worldwide, not every woman is held responsible for producing virginal blood on the heterosexual wedding night. In fact, ‘the sexist social contexts in which women act are also racialized spaces’.^46^ For example, racialized women, especially Muslim women or the ‘sexual subaltern’,^47^ are considered victims of virginity myths and the associated assumption of the need for virginal blood.^48^ Yet, Islam, like other religions, is embodied in multiple ways and women are (not) affected by the need to produce virginal blood for a variety of reasons. But, because sexist social contexts differ,^49^ racialized women are often provided with fewer resources to ‘exercise their intimate capacities’.^50^

This ‘intimate racial injustice’^51^ is also evident in the intertwining of virginal blood with imperialist practices. European imperialism, for instance, feeds from the central idea of ‘racial [blood] purity’,^52^ ensured especially through the control of racialized women’s sexual practices.^53^ For example, in the 1960s and 1970s several South Asian women were subject to virginity tests upon immigration to the UK, reflecting ‘a racism fostered by the former colonialism of the British Commonwealth’.^54^ The virginity myth, however, also serves imperial resistance. In the context of the Soviet-led ‘women’s emancipation politics’,^55^ the enforcement of premarital virginity of Central Asian women marks a patriarchal opposition to the imperial power. The idea of chaste Central Asian women allows for moral differentiation from seemingly unchaste Russian women.^56^

Last, virginal blood is commodified. Virginal blood during the heterosexual wedding night does not only turn into ‘a family’s commodity’^57^ through the family’s lifelong investment in preserving a young woman’s virginity through regulating her sexuality. Virginity is also increasingly medically commodified.^58^ Hymenoplasty, virginity tests,^59^ virginity as ‘preventive public health strategy’^60^ for fighting HIV/Aids and other STDs provoke questions of blood ownership and the marketization of virginity. The ‘geographical specificity’^61^ of the production and economic assessment of blood (products) in blood commodification processes reveals that virginity performances cannot be judged separate from their cultural and socio-economic contexts.

The discussion on how virginal blood functions through various mechanisms of power signals submission to patriarchal, heterosexist, and racist norms through virginity performances. Yet, as I argue in this paper, this view remains limited in terms of what virginal blood can do, for women affected by the performance of virginal blood do not passively endure the violence^62^ of virginity myths. Rather, they resist the regulation of their bodies and sexualities enforced through the virginity myth in multiple ways. They are centrally involved in the construction of intimate justice through subverting gendered and sexist systems of sexuality control,^63^ ‘expand[ing] discursive horizons’^64^ of virginity performances, authoring narratives^65^ of virginal blood and providing resources^66^ regarding virginity myths on Instagram.

Building on and contributing to the rich cultural geographic scholarship on blood, I mobilize virginal blood in this paper as ‘a powerful lens through which to examine minute social processes’^67^ and to examine spatial transformations. The intimate geographies of virginal blood attend to the scalar doings of blood in virginity performances and reveal ways in which the appropriation of virginal blood creates foundations for intimate body justice.

## Accessing virginal blood

My analysis is based on ethnographic research in Kyrgyzstan’s two largest cities Bishkek (976,000 inhabitants) and Osh (256,000 inhabitants) in summer 2017 and 2019 and on the social media platform Instagram since 2018, where I have conducted long-term digital ethnography^68^ with public Russian-language content targeting Kyrgyzstani audiences. The material for this paper stems from my visit to three gynecological practices, plus 23 unstructured and semi-structured interviews with middle-class, able-bodied, ethnically mixed, Kyrgyz, Uzbek, and Russian women (20) and men (3) aged 18–55 in Russian and English, and from Instagram. Fifteen of the interview partners worked in and with local organizations promoting youth and women’s rights. I met eight women who work elsewhere through personal contacts. Most of the interview partners lived in cities and had completed university at the time of our conversation. They thus do not represent the majority population in Kyrgyzstan, because 66% of the population lives in rural areas.^69^ About half of the women I talked to were unmarried, and some sexually active. A few were divorced. The myth of virginity thus affected them to varying degrees. The norm of premarital virginity in Kyrgyzstan^70^ does not necessitate that unmarried women are sexually inactive or will face consequences if not performing virginity.

Bringing up the topic of virginity in research interviews and casual conversations with people in Kyrgyzstan is, however, methodologically challenging. On the one hand, talking about sexuality and the intimate body is a social taboo.^71^ On the other hand, my identity as a female foreigner positioned me as a trusted conversation partner.^72^ I too shared knowledge about my intimate female body and experiences with heterosexual penetration. At the same time, as a race-, body-, and citizenship-privileged guest from Europe, I remained detached from potentially judgmental family communities. My partially detached positionality thus at times also weakened my ability to empathize with women’s sensitive and potentially (re-)traumatizing topic of premarital virginity. Every person I quote in this paper agreed to (anonymously) share their knowledge of and experiences with the virginity myth in Kyrgyzstan.

## The intimate injustices of virginal blood in Kyrgyzstan

‘She just didn’t bleed, that’s all’,^73^ comments Cholpon, a gynecologist from Bishkek, on her colleague Gulzat’s account of the fate of a young woman from her extended family in southern Kyrgyzstan. Because she did not bleed on her wedding night, the groom’s family took the young bride to see a doctor. The latter ‘evidenced’^74^ her assumed non-virginity. ‘It was a scandal’, exclaims Gulzat. ‘They threw her out’.^75^ Almost every person I spoke with in Kyrgyzstan knew a similar story. The interlocutors were not affected themselves, but they mentioned people from their immediate environment.

Even though today, according to the interviewees, many young women living in Bishkek or other major cities in Kyrgyzstan do not have to perform virginity on their wedding night, the public announcement of ‘not bleeding on the wedding night’ has major consequences, especially for women in southern Kyrgyzstan, from ethnic minorities, or from poor families.^76^ For example, if a woman is scandalized, her family of origin might not accept her return; if they do, her public image might remain damaged. Also, the groom’s family can refuse to pay the bride price.^77^ Besides, marking a bride as a non-virgin potentially serves as a reason for the husband or mother-in-law to discriminate against the bride, even years later.^78^

In Kyrgyzstan, the intimate injustices of virginal blood are thus particularly evident in the context of heterosexual marriage. First, there is emotional pressure to marry heterosexually and to fulfill certain bodily expectations in the performance of virginity myths. Second, myths about virginal blood legitimize violent practices such as bride kidnapping. Third, the expectation of virginity performances from unmarried young women indicates the continuation of colonial violence in Kyrgyzstan. Although heterosexual marriage rates are high in Kyrgyzstan^79^ unmarried women are often pushed to marry. The virginity myth can be a tool that turns heterosexual marriage into a form of gendered violence:

*It is the violence to get married [. . .] because she is a virgin [. . .]. It’s like putting this idea in her mind that it will be better to get married to him. It’s not physical violence. It is emotional violence.*^80^

Virginity performances can moreover threaten health, another form of violence. If, as Bermet explains, the groom lacks knowledge about female anatomy, he might use excessive force in sexual penetration to ensure bleeding: ‘Maybe they think, there should be like two liters of blood?!’^81^ In fact, as Kim^82^ reports from her fieldwork:

*“The women [of the groom’s extended family] advised the groom to make sure the bride ‘leaks enough blood’. The groom followed their instructions and when the marriage was consummated the bride passed out. These women brought her to the local hospital where a surgeon examined her. He identified a critical loss of blood, multiple and severe vaginal tears and performed a relevant surgery”.*

Kim classifies this sexual activity in the wedding night as ‘organized rape which is carried out in a collective fashion’^83^ as the groom and the relatives of both the groom and the bride consent to this kind of violence – indeed, they legitimize and encourage it to comply with allegedly traditional norms. In general, successful virginity performances in wedding ceremonies through the production of blood during sexual contact are inherently collective endeavors. They demonstrate, for example, that parents raised their daughter well, or they increase the status of another female family member.

The practice of bride kidnapping is a particularly salient example of the intimate body injustices of virginal blood. *Ala kachuu*, bride kidnapping, that is ‘getting married by abducting a young woman’,^84^ marks a much-debated form of forced marriage in Kyrgyzstan. Both consensual and non-consensual forms have been studied in Central Asia and especially in Kyrgyzstan,^85^ although the practice is also prevalent elsewhere.^86^ Some argue that bride kidnapping marks a ‘reclamation of native “traditions” in post-Soviet republics, others frame it as an outgrowth of economic desperation or weak rule of law’.^87^ For example, consensual bride kidnapping was a way to avoid expensive bride prices during the economically challenging times after independence:

*I have one sister who did that, they made it look like it was ala kachuu. They got in the car and just drove off. It wasn’t a real capture. Just because, during that period, in the 90s, people were very poor. Things were not like kalym [bride price]. Everyone knew they had an agreement.*^88^

Virginal blood becomes an important element for the successful performance of bride kidnapping because ‘when a woman is kidnapped for marriage and enters the groom’s house, she is [. . .] no longer considered to be a virgin’.^89^ Also rape might seal the marriage arrangement to ‘shame her into staying’.^90^ In bride kidnapping, virginal blood might not be relevant in its material form on a blood-stained bedsheet. Rather, the bride abduction signals to the outside community that the woman can no longer prove virginity for another marriage. Whether or not she experienced sexual penetration during the abduction remains secondary. However, the effectiveness of virginity myths remains key because ‘who would kidnap a non-virgin?’^91^

Many scholars and feminist activists understand virginal blood’s complicity in bride kidnapping as an expression of colonial power^92^ and efforts to resist Soviet colonization. Virginity performances are welcomed as a recognition and revival of national identities for ‘dis-identifying with the Soviet Union and establishing new ethno-national sovereignty’.^93^ Soviet authorities themselves probably fueled practices such as bride kidnapping:

*It’s believed that this was a deliberate policy of the Soviet Union to show us natives as wild. Like, look how wild they are, they are stealing people. Although we didn’t have non-consensual kidnapping before. It was done with the girl’s consent. But during Soviet times it was shown as if we practiced violent kachuu, and then, the Soviets came and turned us into humans. They imposed these feelings on us so that later, we thought we had such a tradition.*^94^

Thus, bride abductions are an expression of a global coloniality^95^: on the one hand, disguised by modernity, Soviet powers constructed bride abductions as a barbaric practice to be overcome; on the other hand, the coloniality of bride abductions became visible in the resistance to the Soviet Union, when ethno-nationalist narratives turned kidnappings into a centuries-old virtue precisely because of the Soviets’ rejection of them. Virginity myths and virginal blood that justify bride abductions thus facilitate the continuation of the global coloniality of intimate gendered, sexualized, and racialized bodies.

Virginal blood becomes a core issue of intimate body injustice in Kyrgyzstan because the virginity myth as a form of gender-based and sexual violence seems protected by law. Although people involved in bride kidnapping now face up to 10 years in prison, rates for forced marriages remain high in Kyrgyzstan.^96^ Feminist and women’s rights activists see reasons for this in a lack of law enforcement. For example, of 895 cases of bride abduction reported to the police between 2013 and 2018, only 168 cases were carried to court.^97^

## Recovering intimate body spaces

Given the lack of state, institutional, and, in some cases, family protections against the violence inherent in virginity performances, affected women and their allies use multiple strategies to deal with the intimate injustices of virginal blood. Brides can reclaim the intimate space of their wedding night through controlling how they produce virginal blood. Or women also refuse to adhere to the virginity norm all together.

While hymenoplasty – the sewing together of vaginal corona tissue a few days before the wedding night to ensure bleeding during penetration – is becoming more common to guarantee successful virginity performances,^98^ interviewees also mentioned less costly and bodily demanding acts of virginal blood production. For example, Gulzat^99^ recounts how brides – often together with the grooms – splash liquid pink-colored vitamin B12 on the bedsheet while relatives outside the door await the ‘proof’^100^ of the bride’s virginity. In other cases, brides ‘just cut their finger and spill the blood, just to calm down their relatives’.^101^ Women and their partners produce blood on the marital linen to be publicly displayed among relatives and sometimes even entire communities.

Thus, many of the people affected by the virginity myth fulfill the expectations of the family because virginity in connection with marriage ‘is not a private thing, it’s public’.^102^ As Nurgul Zhanabayeva emphasizes, young people ‘feel responsibility for preserving the honor of their families’^103^ through evidencing virginity through blood. Producing virginal blood in the wedding night becomes the visceral seal to hold the intimate unjust social order in place and to keep virginity performances in public space.

Through defining *how* they perform virginity, independent of their sexual experiences prior to and/or outside marriage, women reclaim control over the intimate space of their sexual bodies. They ‘adopt an oppositional attitude toward social norms’^104^ by rescaling the production site of virginal blood, from virginal blood submitted to community surveillance to virginal blood subverting intimate body injustices. Producing virginal blood in one’s own way becomes a mode of

*gender tricksterism as a way of transcending the limitations of colonialist, orientalist, ideological, cultural, religious, ethnic, and sexual nature, and a way of acting beyond and around the dominant power structures, weakening them from a particular gendered exteriority.*^105^

A more radical form of reclaiming one’s intimate body space is the refusal of performing the virginity myth. Among urban Kyrgyz youth, it is socially accepted that not every romantic relationship results in heterosexual marriage.^106^ Especially if, the couple restrained from premarital penetrative sex that could jeopardize the virginity performance on the wedding night. But, even a woman’s sexual activity before marriage does not automatically determine whether they will choose to perform virginity in their future wedding night. Zara explains:

*I had my first sex when I got married according to Islamic tradition, when I was almost 18. My parents know about it and for them it’s OK. But, my old relatives, like my grandmother and grandaunt, they tell me to find a boyfriend and to get married soon. [. . .] And they tell me, to go to a clinic to do the operation [hymenoplasty] before I will get married [again]. But, no, this is not for me.*^107^

Despite pressure from relatives, Zara explains that hymenoplasty is out of question for her. Zara refuses to comply with the norm to produce virginal blood in the wedding night and thus ‘expand[s] discursive horizons regarding [. . .] female sexual desire’.^108^ She both openly shares with her family members that she was sexually active but that she has no intentions to comply with virginity norms again.

Her refusal to perform virginity as socially expected, however, requires emotional work. Zara’s active involvement at a queer feminist youth organization in Osh helped her cope with feelings of shame and insecurity related to her future nonperformance of virginity. She co-designed a project on virginity myths which involved knowledge production about sexual moralities and female anatomy and the sharing of marginalized sexual experiences. Gradually this engagement helped her dismantle her ‘own stereotypes about sex and virginity’^109^ and wipe off the insecurity and shame about her sexual self.

Both people who perform virginity in self-conscious ways and people who refuse to adhere to virginity norms, build the foundations for intimate body justice in Kyrgyzstan. Through subverting virginity performances and expanding discursive horizons regarding women’s sexual activities in Kyrgyzstan, they reveal strategies of resistance in a societal context that seeks to regulate their intimate body parts in public spaces. They demonstrate that their intimate bodies are ‘plurally capable’^110^ and of more than restraining from sexual activities and enduring penetrative sex that leaks ‘enough blood’.^111^ They disclose powerful intimate body capacities through controlling and/or refusing the production of virginal blood and the shame connected to women’s premarital sexual practices. The normalization of moral support and non-interference from family members, like parents, is crucial in strengthening these foundations of intimate body justice.

## Developing digital spaces for intimate body knowledge

Centrally, young women’s ‘intimate-sphere resistance efforts’^112^ are strengthened through digital spaces. Social media platforms enable space for authoring^113^ different virginal blood narratives and provide resources^114^ on intimate body knowledge. For example, virginal blood appears on Instagram in various forms. As a social media platform centered on the visual,^115^ Instagram allows virginal blood to appear in a visual format. This is particularly noteworthy because although the aim of many virginity performances is to have visual evidence of a bride’s chastity with virginal blood on a white cloth, this moment is not usually captured in aesthetic images like other wedding moments. Virginal blood on the sheet remains a byproduct of a successful virginity performance, although essential value is attached to the presence of blood on the wedding night.

**Figure 1.**
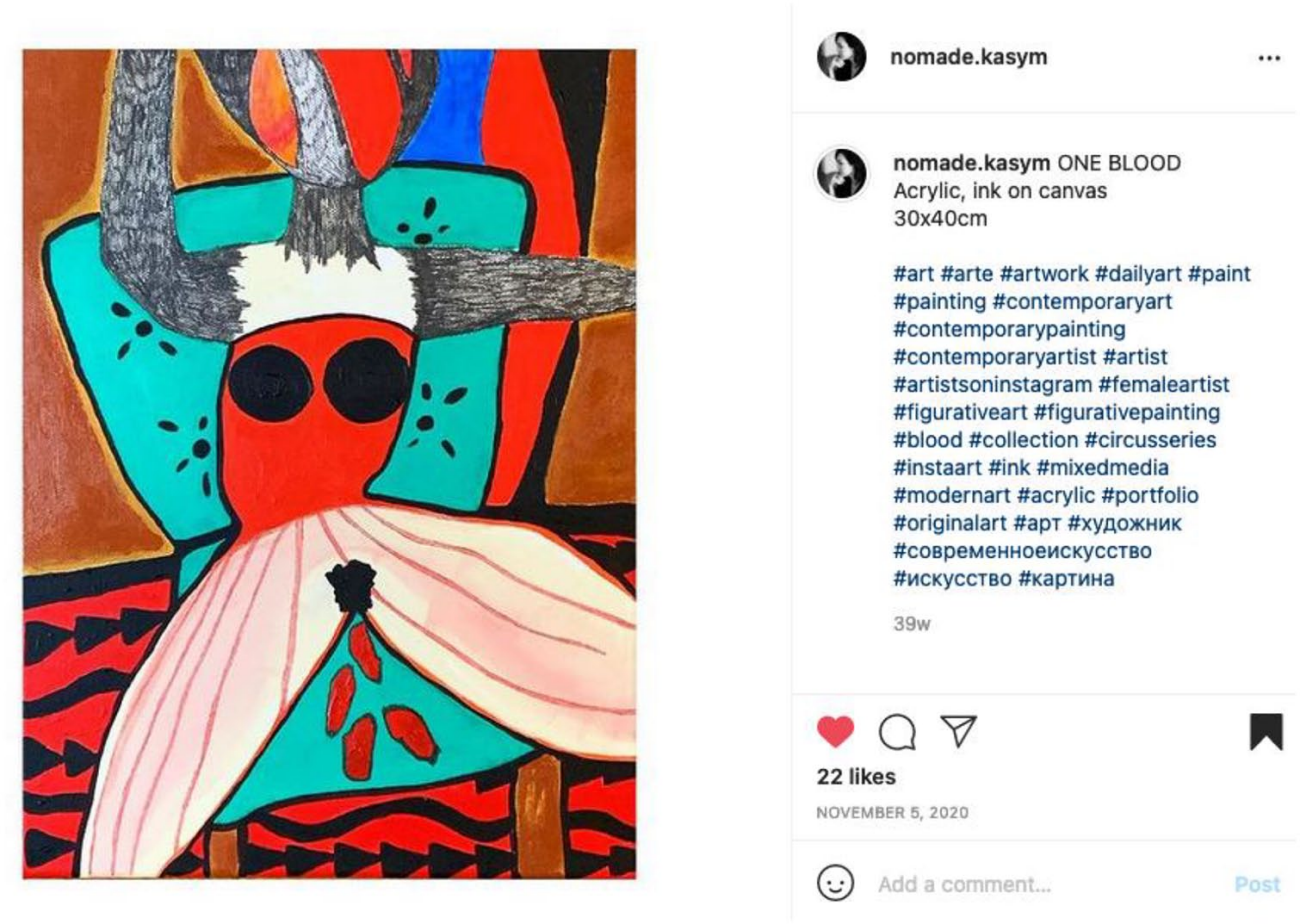
Instagram post by @nomade.kasym entitled ONE BLOOD on November 5, 2020, reprinted with permission (photo taken by author).

In the image ONE BLOOD (Figure 1) by artist Aika Kasym, virginal blood gets a visual stage on Instagram. Drawing attention to the taboo and stigmatization of vaginal blood in general, through her artwork Kasym encourages readers to ‘cherish and appreciate’^116^ any blood that comes from the vagina. Her artwork tells a visual story about virginal blood that hints at the ‘constraints on the aspects of the body [. . .] most closely associated with femininity’.^117^

Instagram has become a space that makes bodily experiences with virginal blood tangible. For example, young people turn to semi-anonymous Instagram forums to educate themselves about virginity performances. Popular accounts such as molodye_mamochki_kg (145,000 followers) or kelinkii.kg (56,700 followers)^118^ offer the opportunity to anonymously ask sensitive questions via direct messaging, which are then posted and opened for comments. For example, a post from 21 February 2018 shared:

*Hello admin!!! Please post! Anonymously. Help me, I am in a difficult situation! [. . .] I have a boyfriend [. . .]. I slept with him. He promises to leave her and to marry [me]. I believe in it. But I am also a bit afraid and have doubts that he will stay [with her]. Blood tells what we do in the wedding night. I am afraid of my parents. I can’t find my place; I am thinking all the time and cry. Come, give me advice!*^119^

Vague ideas about virginal blood expectations in the wedding night coupled with fear and insecurity push this author to seek advice on Instagram. Even if at times the writing remains unclear, the fear of the blood performance in the potential wedding night comes through. The post reveals the vulnerability and emotional stress resulting from violent virginity expectations. It also shows how a digital public space unfolding through Instagram allows people to author virginal blood narratives, to anonymously share tabooed sexual experiences and to create room for uncomfortable emotions such as fear and insecurity. Activists like Guliaim Greeny and Uluk Batyrgaliev, however, complain about a lack of safety and quality control in these semi-anonymous forums. Oftentimes any Instagram user can comment on these posts and thus potentially expose the post’s author to further emotional stress.^120^

A lack of knowledge around virginal blood is inspiring the development of Instagram-based sexual education and the provision of qualitative resources for intimate body knowledge. For example, in February 2020, Zara published a post on Instagram featuring her smiling portrait and the hashtag #девственность [#virginity] to address the violence of virginal blood in a longer text:

*The hymen has long been considered a “sign of chastity” and innocence in girls. Strange, isn’t it? It’s not just girls who have sex, after all ). That’s not all, it’s the hymen itself that must burst during the first penetrative sex, accompanied by blood and pain. Imagine this process. Brrrrr 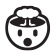. . .*^121^

Against the backdrop of absent or stigmatized formal sex education, queer feminist activists like Zara are taking on the task of addressing taboo sexual topics and educating about myths surrounding virginity performances. Posts like Zara’s on Instagram bring together people who are faced with the social expectation of virginity performances coupled with inexperience, insecurity, and questions about virginal blood. Of Kyrgyzstan’s population of 6.68 million, 2.95 million, or ‘86.5 percent of the local internet user base’^122^ use Instagram, giving these posts potentially enormous reach. In fact, sexual peer educator Batyrgaliev confirms that sex education addressing virginity issues mainly takes place on Instagram these days.^123^ Drawing on his knowledge and reflections of 10 years of sexual and reproductive rights activism in Kyrgyzstan, he started blogging about ‘sex, reproductive health, sexual relationships [. . .] and more’^124^ on Instagram in fall 2020 as @batyrgaliev_ (10,000 followers). He shares short videos that show him, for example, discussing contraception, and invites followers to anonymously answer questions through Instagram’s story function. Like a few other accounts, Batyrgaliev aims to normalize intimate body knowledge and sex talk in Kyrgyzstan’s digital public spaces.^125^ Virginity is a recurring theme mainly because users ask for advice regarding virginity and virginal blood performances in direct messages.

Virginal blood creates new digital spaces for marginalized sexual knowledge and experiences on Instagram precisely because the platform offers anonymity, reach beyond personal networks and multi-sensory communication. Tabooed sexual experiences are finding space online through narratives authored by people affected by the virginity myth. These narratives make visible ‘the constraints the system place[s] on [their] ability to make choices regarding sexual relations’.^126^ The provision of knowledge resources about the virginity myth and virginal blood through Instagram do not just demystify intimate body processes, but create foundations for intimate body justice in Kyrgyzstan through supporting women’s bodily integrity.^127^

## Toward intimate body justice in Kyrgyzstan

This article traces foundations for intimate justice in Kyrgyzstan in the context of the virginity myth, following Threadcraft’s deeply geographic intimate justice project.^128^ Following her call, intimate justice in the context of globally circulating virginity myths means changing intimate geographies of the body and its parts. By each person feeling and exploring their own sexualities and intimate body in safe spaces, bodily feelings and sexualities can grow and develop independent of societal constraints such as virginity myths and without shame and fear regarding sexual experiences and practices.

Because virginal blood signifies the power of virginity myths, I followed the intimate geographies of virginal blood to uncover basic elements for intimate body justice. Virginal blood does not only reveal the inherent intimate injustice of virginity myths. Precisely, the volatility and uncertainty of virginal blood offers possibilities to reset bodily boundaries and generate new spaces for intimate body experiences and knowledge. The self-determined production of virginal blood on the wedding night (e.g. finger cuts), the withdrawal from virginity performances (e.g. refusing hymenoplasty), and the relocation of narratives and resources around virginity and virginal blood into digital space (e.g. on semi-anonymous Instagram forums) reveal that people resist virginity myths’ intimate injustice through employing multiple strategies to appropriate virginal blood.

Virginal blood’s complicity in gendered, sexualized, racialized, postcolonial, and commodified power relations, makes clear that virginal blood operates on multiple scales. Virginal blood is not a random and/or passive byproduct of heterosexuality performances. Rather, virginal blood functions as the visceral seal to legitimate the heterosexual society and its inherent gender-based and sexualized violence based on powerful entanglements of family, nationalist, and colonial ideas. At the same time, virginal blood transgresses bodily boundaries and enables the practicing of resistance to intimate injustice on social media, where people experience closeness and trust precisely because they can remain anonymous and at physical distance.

Achieving intimate body justice in Kyrgyzstan regarding virginity myths will require the recognition of the diversity of people’s needs and desires to be sexually active, to marry, and/or to perform virginity myths. Each person should be able to decide how to deal with virginity myths and, if relevant, the publicity or privacy and techniques of their virginity performance. Individual bodies and their bodily capabilities also need to be detached from ethno-nationalist myths. In the context of Kyrgyzstan, this includes, above all, acknowledging the colonial heritage behind virginity myths. Furthermore, fulfilling virginity performances requires recognition as physical and emotional work that relies on certain resources such as support and trust from family and community members. Finally, access to comprehensive sexual education and intimate body knowledge would empower people to make their own decisions about virginity representations. When this intimate body knowledge emerges through the co-constitution of analog and digital experiences and practices, it requires people to be digitally literate and have safe access to digital spaces.

By analyzing global and multi-scalar entanglements of body parts and the emergence and transformation of intimate spaces, geographers can significantly contribute to the exploration of spatial capacities of different body parts. This requires, however, taking the spatial reach of body parts seriously and not overlooking supposedly small and/or ephemeral body parts such as virginal blood, for it is a spatially relevant object of research that points to global colonialities, the claiming of intimate body spaces, and ‘intimate-sphere resistance’^129^ in digital spaces.

## Notes

1. Bermet, Personal conversation on 18.08.2017 in Osh (translation by author).

2. To protect the privacy of research partners, first names mentioned are pseudonyms.

3. F.Abdilashimova, *Reconstructing Virginity: Hidden Sentiments And Complexities Behind Hymenoplasty in Kyrgyzstan* (Budapest: Central European University, 2020); J.Droeber, ‘Back to Islam? Women’s Sexualities and Body-Politics in Muslim Central Asia’, *Hawwa*, 4, 2006, pp. 181–213; E.Kim, *Violence Against Women and Girls. Gender in Society Perceptions Study (GSPS) of the Kyrgyz Republic* (Bishkek: UN Women, 2018); D.T.Kudaibergenova, ‘Project Kelin: Marriage, Women, and Re-Traditionalization in Post-Soviet Kazakhstan’, in M.Najafizadeh and L.L.Lindsey (eds), *Women of Asia* (New York: Routledge, 2018), pp. 379–90.

4. C.Harris, ‘State Business: Gender, Sex and Marriage in Tajikistan’, *Central Asian Survey*, 30, 2011, pp. 97–111.

5. See E.Kim, ‘Re-feminizing the Post-Soviet Women: Identity, Politics and Virginity Ceremonies in Contemporary Kyrgyzstan’, *Journal of Gender Studies*, 29, 2020, pp. 706–16, p. 706.

6. K.Milles, J.Holmdahl, I.Melander and K.Fugl-Meyer, ‘“Something That Stretches During Sex”: Replacing the Word Hymen With Vaginal Corona to Challenge Patriarchal Views on Virginity’, *Gender and Language*, 12, 2018, pp. 294–317.

7. Virginity myths also impact people who do not identify as women (e.g., trans men, non-binary people assigned female at birth). Yet, as virginity myths are closely linked to ideas about how women should perform their gender and sexual identity, the article focuses on the experiences of woman-identifying people.

8. See for example A.Ahmadi, ‘Recreating Virginity in Iran: Hymenoplasty as a Form of Resistance’, *Medical Anthropology Quarterly*, 30, 2016, pp. 222–37; G.Myrzaeva, ‘Women in Kyrgyzstan Challenging Stereotypes and Virginity Tradition’, 2011.

9. N.Oswin and E.Olund, ‘Governing Intimacy’, *Environment and Planning D: Society and Space*, 28, 2010, pp. 60–7; G.Pratt and V.Rosner, ‘Introduction: The Global & the Intimate’, *Women’s Studies Quarterly*, 34, 2006, pp. 13–24; G.Valentine, ‘The Ties That Bind: Towards Geographies of Intimacy’, *Geography Compass*, 2, 2008, pp. 2097–110; P.Vasudevan, ‘An Intimate Inventory of Race and Waste’, *Antipode*, 53, 2021, pp. 770–90.

10. E.Hall, ‘“Blood, Brain and Bones”: Taking the Body Seriously in the Geography of Health and Impairment’, *Area*, 32, 2000, pp. 21–9; K.Ramdas, ‘Is Blood Thicker Than Water? Single Indian Singaporean Women and the Geographies of “Being” Family’, *Gender, Place & Culture*, 22, 2015, pp. 255–70; S.Sodero, ‘Vital Mobilities: Circulating Blood Via Fictionalized Vignettes’, *cultural geographies*, 24, 2019, pp. 109–25.

11. S.Threadcraft, *Intimate Justice: The Black Female Body and the Body Politic* (Oxford: Oxford University Press, 2016), p. 33.

12. Threadcraft, *Intimate Justice*, p. 6.

13. Threadcraft, *Intimate Justice*, p. 33.

14. D.Cindoglu, ‘Virginity Tests and Artificial Virginity in Modern Turkish Medicine’, *Women’s Studies International Forum*, 20, 1997, pp. 253–61; S.Gupta, ‘The Unhealthy Fixation With Virginity: Pre-Marital Sex, Hymen & Hymenoplasty’, 2017; F.Mernissi, ‘Virginity and Patriarchy’, *Women’s Studies International Forum*, 5, 1982, pp. 183–91.

15. S.Abboud, L.S.Jemmott and M.S.Sommers, ‘“We Are Arabs:” The Embodiment of Virginity Through Arab and Arab American Women’s Lived Experiences’, *Sexuality & Culture*, 19, 2015, pp. 715–36, p. 716.

16. J.Kelso, ‘Irigaray’s Madonna’, *Feminist Theology*, 23, 2015, pp. 171–85, p. 175.

17. Mernissi, ‘Virginity and Patriarchy’, p. 191.

18. S.S.Chisale, ‘Decolonising HIV Prevention: A Critical Examination of Ukusoma and Virginity Testing’, *AlterNation*, 23, 2016, pp. 217–35, p. 225.

19. A.McClintock, *Imperial Leather: Race, Gender and Sexuality in the Colonial Contest* (New York: Routledge, 1995), p. 30.

20. S.Skandrani, T.Baubet, O.Taïeb, D.Rezzoug and M.R.Moro, ‘The Rule of Virginity Among Young Women of Maghrebine Origin in France’, *Transcultural Psychiatry*, 47, 2010, pp. 301–13, p. 306.

21. L.L.Wynn and S.Hassanein, ‘Hymenoplasty, Virginity Testing, and the Simulacrum of Female Respectability’, *Signs: Journal of Women in Culture and Society*, 42, 2017, pp. 893–917, p. 895.

22. A.Ahmadi, ‘Hymenoplasty in Contemporary Iran: Liminality and Embodiment of Contested Discourses’, in L.L.Wynn and A.M.Foster (eds), *Abortion Pills, Test Tube Babies, and Sex Toys: Emerging Sexual and Reproductive Technologies in the Middle East and North Africa* (Nashville: Vanderbilt University Press, 2016), pp. 145–58.

23. A.Parla, ‘The “Honor” of the State: Virginity Examinations in Turkey’, *Feminist Studies*, 27, 2001, pp. 65–88, p. 66.

24. S.Moslener, *Virgin Nation: Sexual Purity and American Adolescence* (Oxford: Oxford University Press, 2015), p. 4.

25. Kim, ‘Re-feminizing the Post-Soviet Women’, p. 712.

26. Kim, ‘Re-feminizing the Post-Soviet Women’, p. 711.

27. Threadcraft, *Intimate Justice*, p. 43.

28. Pratt and Rosner, ‘Introduction’.

29. S.H.Smith, *Intimate Geopolitics: Love, Territory, and the Future on India’s Northern Threshold* (New Brunswick: Rutgers University Press, 2020).

30. S.Chattopadhyay, ‘Borders Re/make Bodies and Bodies Are Made to Make Borders: Storying Migrant Trajectories’, *ACME: An International Journal for Critical Geographies*, 18, 2019, pp. 149–72.

31. E.Militz, ‘Killing the Joy, Feeling the Cruelty: Feminist Geographies of Nationalism in Azerbaijan’, *Environment and Planning C: Politics and Space*, 38, 2020, pp. 1256–74.

32. E.Nikiforova and O.Brednikova, ‘On Labor Migration to Russia: Central Asian Migrants and Migrant Families in the Matrix of Russia’s Bordering Policies’, *Political Geography*, 66, 2018, pp. 142–50.

33. Valentine, ‘The Ties That Bind’, p. 2103.

34. Feminist Geographies of Intimacy Course Handbook 2017, cited in S.Burke, A.Carr, H.Casson, K.Coddington, R.Colls, A.Jollans, S.Jordan, K.Smith, N.Taylor and H.Urquhart, ‘Generative Spaces: Intimacy, Activism and Teaching Feminist Geographies’, *Gender, Place & Culture*, 24, 2017, pp. 661–73, p. 662.

35. A.Mountz, ‘Political Geography III: Bodies’, *Progress in Human Geography*, 42, 2018, pp. 759–69, p. 759.

36. See R.Colls, ‘Materialising Bodily Matter: Intra-Action and the Embodiment of “Fat”’, *Geoforum*, 38, 2007, pp. 353–65; R.Colls and M.Fannin, ‘Placental Surfaces and the Geographies of Bodily Interiors’, *Environment and Planning A: Economy and Space*, 45, 2013, pp. 1087–104; C.V.Faria and H.Jones, ‘A Darling^®^ of the Beauty Trade: Race, Care, and the Imperial Debris of Synthetic Hair’, *cultural geographies*, 27, 2020, pp. 85–99.

37. R.Longhurst, *Bodies: Exploring Fluid Boundaries* (London: Routledge, 2001).

38. A.-M.Farrell, *The Politics of Blood: Ethics, Innovation and the Regulation of Risk* (Cambridge: Cambridge University Press, 2012), p. 3.

39. C.Nash, ‘Geographies of Relatedness’, *Transactions of the Institute of British Geographers*, 30, 2005, pp. 449–62.

40. Farrell, *The Politics of Blood*.

41. B.Parry, ‘Entangled Exchange: Reconceptualising the Characterisation and Practice of Bodily Commodification’, *Geoforum*, 39, 2008, pp. 1133–44, p. 1135; see also M.Fannin, ‘Personal Stem Cell Banking and the Problem With Property’, *Social and Cultural Geography*, 12, 2011, pp. 340–56.

42. See for example J.Kent, M.Fannin and S.Dowling, ‘Gender Dynamics in the Donation Field: Human Tissue Donation for Research, Therapy and Feeding’, *Sociology of Health & Illness*, 41, 2019, pp. 567–84; J.Kent and A.-M.Farrell, ‘Risky Bodies in the Plasma Bioeconomy: A Feminist Analysis’, *Body & Society*, 21(1), 2014, 29–57; Sodero, ‘Vital Mobilities’.

43. Myrzaeva, ‘Women in Kyrgyzstan’.

44. Ramdas, ‘Is Blood Thicker Than Water?’, p. 257.

45. Nash, ‘Geographies of Relatedness’, p. 450.

46. Threadcraft, *Intimate Justice*, p. 65.

47. A.Roy, ‘Spivak Alert! Conceiving Sexuality Through a Subaltern Lens’, *Gender, Place & Culture*, 28, 2021, pp. 208–23, p. 1.

48. See L.Abu-Lughod, ‘Do Muslim Women Really Need Saving? Anthropological Reflections on Cultural Relativism and Its Others’, *American Anthropologist*, 104, 2002, pp. 783–90.

49. Threadcraft, *Intimate Justice.*

50. Threadcraft, *Intimate Justice*, p. 66.

51. Threadcraft, *Intimate Justice*, p. 142.

52. McClintock, *Imperial Leather*, p. 48.

53. See also R.Mawani, ‘“Half-Breeds,” Racial Opacity, and Geographies of Crime: Law’s Search for the “Original” Indian’, *cultural geographies*, 17, 2010, pp. 487–506.

54. E.Smith and M.Marmo, ‘Uncovering the “Virginity Testing” Controversy in the National Archives: The Intersectionality of Discrimination in British Immigration History’, *Gender & History*, 23, 2011, pp. 147–65, p. 162.

55. Kim, ‘Re-feminizing the Post-Soviet Women’, p. 708.

56. M.Tlostanova, *Gender Epistemologies and Eurasian Borderlands* (New York: Routledge, 2010).

57. A.Titova, «Девственность — товар семьи». Как кыргызских девушек стыдят из-за стереотипов о «непорочности» [‘“Virginity - A Family’s Commodity”. How Kyrgyz Girls Are Shamed Because of Stereotypes About “Purity”’], 2019 (translation by author).

58. Ahmadi, ‘Recreating Virginity in Iran’.

59. See also E.Durojaye, ‘The Human Rights Implications of Virginity Testing in South Africa’, *International Journal of Discrimination and the Law*, 16, 2016, pp. 228–46.

60. A.Wickström, ‘Virginity Testing as a Local Public Health Initiative: A “Preventive Ritual” More Than a “Diagnostic Measure”’, *Journal of the Royal Anthropological Institute*, 16, 2010, pp. 532–50, p. 234; see also Chisale, ‘Decolonising HIV Prevention’.

61. Parry, ‘Entangled Exchange’, p. 1144.

62. Kim, ‘Re-feminizing the Post-Soviet Women’.

63. See Threadcraft, *Intimate Justice*, p. 129.

64. Threadcraft, *Intimate Justice*, p. 112.

65. See Threadcraft, *Intimate Justice*, p. 41ff.

66. See Threadcraft, *Intimate Justice*, p. 144.

67. J.Carsten, *Blood Work. Life and Laboratories in Penang* (Durham and London: Duke University Press, 2019), p. 16.

68. G.de Seta, ‘Three Lies of Digital Ethnography’, *Journal of Digital Social Research*, 2, 2020, pp. 77–97.

69. United Nations, *2019 Demographic Yearbook. Annuaire démographique*. 70th issue/70e ed. (New York: United Nations, 2020), p. 155.

70. Kim, ‘Re-feminizing the Post-Soviet Women’.

71. Z.Syrgak kyzy, *Exploring the Discourses About Sexual Education in Kyrgyzstan: The Case of Government, Non-Government and Political Institutions* (Bishkek: American University of Central Asia, 2014).

72. See for a similar experience Ahmadi, ‘Recreating Virginity in Iran’, p. 227.

73. Cholpon, Personal conversation on 08.08.2017 in Bishkek (translation by author).

74. Gulzat, Personal conversation on 08.08.2017 in Bishkek (translation by author).

75. Gulzat, Personal conversation.

76. For example N.Smankulova, Personal conversation on 12.08.2017 in Bishkek; Aiperi, Personal conversation on 06.08.2017 in Bishkek (translation by author); B.Talasbek kyzy, Personal conversation on 06.06.2019 in Bishkek; Zara, Personal conversation on 13.06.2019 in Osh (translation by author).

77. Titova, ‘Virginity - A Family’s Commodity’.

78. Gulzat, Personal conversation.

79. United Nations, *2019 Demographic Yearbook*.

80. Smankulova, Personal conversation.

81. Bermet, Personal conversation.

82. Kim, ‘Re-feminizing the Post-Soviet Women’, p. 710.

83. Kim, ‘Re-feminizing the Post-Soviet Women’, p. 710.

84. As defined in a Kyrgyz dictionary and quoted in R.Kleinbach and L.Salimjanova, ‘Kyz ala kachuu and adat: Non-Consensual Bride Kidnapping and Tradition in Kyrgyzstan’, *Central Asian Survey*, 26, 2007, pp. 217–33, p. 218.

85. See for example A.Baibolova, “What Will People Say?” Bride Kidnapping in Kyrgyzstan—What Makes a Woman Stay, 2019; A.Sadykova, *The Role of Women Facilitators of Bride Kidnapping in Kyrgyzstan* (Manhattan: Kansas State University, 2021); B.Sataeva, *Public Shaming and Resistance in the Context of the Bride Kidnapping Phenomenon in Kyrgyzstan* (Utrecht: University of Utrecht, 2017).

86. C.Werner, C.Edling, C.Becker, E.Kim, R.Kleinbach, F.E.Sartbay and W.Teachout, ‘Bride Kidnapping in Post-Soviet Eurasia: A Roundtable Discussion’, *Central Asian Survey*, 37, 2018, pp. 582–601.

87. Werner et al., ‘Bride Kidnapping in Post-Soviet Eurasia’, p. 583.

88. G.Greeny, Personal conversation on 06.06.2019 in Bishkek (translation by author).

89. Sataeva, *Public Shaming and Resistance in the Context of Bride Kidnapping*, p. 27.

90. Kim, *Violence Against Women and Girls*, p. 18.

91. E.Kim, Personal conversation on 09.07.2019 in Bishkek.

92. E.Kim and E.Molchanova, ‘Resurgence of Ethnic Sports in Post-Soviet Kyrgyzstan: Still Hunting for Women’, in G.Molnar, S.N.Amin and Y.Kanemasu (eds), *Women, Sport and Exercise in the Asia-Pacific Region* (London: Routledge, 2018), pp. 49–60; Kim, *Violence Against Women and Girls*; Kleinbach and Salimjanova, ‘Kyz ala kachuu and adat’.

93. Kim, ‘Re-feminizing the Post-Soviet Women’, p. 707.

94. Greeny, Personal conversation.

95. M.V.Tlostanova and W.D.Mignolo, ‘Global Coloniality and the Decolonial Option’, *Kult*, 6, 2009, pp. 130–47.

96. G.F.Fillo and A.Negruta, *Keep the Promise, Accelerate the Change. Taking Stock of Gender Equality in Europe and Central Asia 25 Years After Beijing* (Istanbul: UN Women, 2020), p. 22.

97. K.Eshaliyeva, ‘Why Domestic Violence is Flourishing in Kyrgyzstan - And How It Could Stop’, 2020.

98. Abdilashimova, *Reconstructing Virginity*; Ahmadi, ‘Recreating Virginity in Iran’.

99. Gulzat, Personal conversation.

100. Bermet, Personal conversation.

101. Aiperi, Personal conversation

102. Aiperi, Personal conversation

103. N.Zhanabayeva, *Restrained by Uyat [Shame]: Culture of Dating and Romantic Relationships Among Urban Kyrgyz Youth* (Astana: Nazarbayev University, 2018), p. 27.

104. Threadcraft, *Intimate Justice*, p. 129.

105. Tlostanova, *Gender Epistemologies and Eurasian Borderlands*, p. 66.

106. Zhanabayeva, *Restrained by Uyat [Shame]*.

107. Zara, Personal conversation.

108. Threadcraft, *Intimate Justice*, p. 112.

109. Zara, Personal conversation.

110. Threadcraft, *Intimate Justice*, p. 32.

111. Kim, ‘Re-feminizing the Post-Soviet Women’, p. 710.

112. Threadcraft, *Intimate Justice*, p. 43.

113. See Threadcraft, *Intimate Justice*, p. 41ff.

114. See Threadcraft, *Intimate Justice*, p. 144.

115. T.Leaver, T.Highfield and C.Abidin, *Instagram. Visual Social Media Cultures* (Cambridge and Medford: Polity Press, 2020).

116. A.Kasym, Direct messaging on Instagram since 5 August 2021.

117. Threadcraft, *Intimate Justice*, p. 50.

118. Through the abbreviation “kg” included in the names of these accounts, they explicitly target a Kyrgyzstan-based audience.

119. molodye_mamochki_kg, Здравствуйте, админ!!! Пожалуйста опубликуйте! Анон. Помогите, я. . ., 2018 (translation by author).

120. G.Greeny, Personal conversation; U.Batyrgaliev, Personal conversation on 03.07.2019 in Bishkek.

121. Zara, #девственность. Пора наверное поговорить о самом объвеянном ужасающими мифами. . ., 2020 (translation by author).

122. S.Kemp, ‘Digital 2022: Kyrgyzstan’, 2022.

123. Batyrgaliev, Personal conversation; see also Abdilashimova, *Reconstructing Virginity*.

124. U.Batyrgaliev, Всем привет  и добро пожаловать на мою страницу. Я буду. . ., 2020 (translation by author).

125. U.Batyrgaliev, Personal conversation on 24.03.2022 via MS Teams.

126. Threadcraft, *Intimate Justice*, p. 41.

127. See Threadcraft, *Intimate Justice*, p. 164.

128. Threadcraft, *Intimate Justice*.

129. Threadcraft, *Intimate Justice*, p. 43.

